# Data science without borders: bridging the divide in data science capacity across African health institutions

**DOI:** 10.3389/fpubh.2025.1695907

**Published:** 2025-12-05

**Authors:** Agnes N. Kiragga, Samuel Iddi, Abel W. Walekhwa, Miranda Barasa, Steve Cygu, Rachel Odhiambo, Moctar Gningue, Aminata Mboup, Anicet Onana, Bethlehem Adnew, Akililu Alemu Ashuro, Simon Hudson, Jay Greenfield, Jim Todd, Tathagata Bhattacharjee, Malvika Sharan, Raphael Sonabend, Damazo Kadengye, Bertrand Hugo Mbatchou, Alemseged Abdissa, Moussa Sarr, Joyce Nakatumba Nabende, Moses Bamutura, Bekure Tamurat, Nebiyu Dereje, Elvis Temfack

**Affiliations:** 1 African Population and Health Research Center, Nairobi, Kenya; 2 Department of Statistics and Acturial Science, University of Ghana, Legon, Accra, Ghana; 3 Disease Dynamics Unit, Department of Veterinary Medicine, University of Cambridge, Cambridge, United Kingdom; 4 Duala General Hospital, Douala, Cameroon; 5 Armauer Hansen Research Institute (AHRI), Addis Ababa, Ethiopia; 6 Committee on Data (CODATA), Paris, France; 7 London School of Hygiene and Tropical Medicine, London, United Kingdom; 8 OSPO Now, London, United Kingdom; 9 Department of Computer Science, Center for Artificial Intelligence Lab, Makerere University, Kampala, Uganda; 10 Africa Centres for Diseases Control and Prevention, Addis Ababa, Ethiopia

**Keywords:** data governance, federated infrastructure, capacity building, African health informatics, cross-institutional collaboration

## Abstract

**Background:**

Effective public health data science in Africa requires a comprehensive understanding of institutional capabilities across multiple dimensions. This study conducted a multidimensional assessment of three African health institutions to examine the availability of health data, healthcare training, data governance needs, and infrastructure capabilities, to inform the use of data science tools to address health challenges.

**Methods:**

The study is a baseline assessment for the Data Science Without Borders Project—a three-year multi-country project implemented in three African health institutions: the Institute for Health Research, Epidemiological Surveillance and Training (IRESSEF) in Senegal, Armauer Hansen Research Institute (AHRI) in Ethiopia, and the Douala General Hospital (DGH) in Cameroon. We designed a baseline structured needs assessment survey to assess: (1) health data availability across sixteen (16) dataset categories; (2) training needs across seven (7) domains, data governance considerations; and (3) infrastructure capabilities, including computing resources, connectivity, and service availability. We then conducted an integrated analysis to identify patterns, gaps, and opportunities across various dimensions, informing project implementation.

**Results:**

The assessment revealed different institutional profiles with complementary strengths and limitations, which are critical for the effective use of data science tools. IRESSEF demonstrated rich data resources (particularly in genomics, maternal health, and geographical health differences), moderate infrastructure limitations (8GB RAM, 67% service capability), and high training needs (data & analytics: 4.7/5.0, data governance: 4.0/5.0). AHRI exhibited superior computing resources (512GB RAM, 64 CPU cores), specialized surveillance data (9.9%), and moderate training needs (average: 3.0/5.0). DGH demonstrated focused strengths in infectious disease research (3.3%), moderate computing resources (32 GB RAM), and large opportunities to use electronic health records for research. Common priorities across institutions included the need for enhancing data & analytical capabilities (average: 4.3/5.0) and use of advanced [artificial intelligence and machine learning analysis techniques (IRESSEF: 5.0, AHRI: 4.0, DGH: 5.0)], and very importantly, the need to establish data governance structures to increase the ability and capacity of the partners to share data for consortium collaborative analyses across Africa.

**Conclusion:**

Our integrated assessment suggests that effective capacity building requires moving beyond standardized approaches to embrace a phased model that leverages institutional needs and complementarities. We recommend: (1) establishing robust data governance frameworks as a foundation; (2) implementing a phased and customized approach where institutions receive training according to their immediate demands and strengths; (3) addressing critical infrastructure gaps to support data. We are involved in science projects in Africa that support federated analyses to maintain data sovereignty. This approach offers potential for a varying African approach to health data science, which could extend to AI adoption and broader continental collaboration.

## Introduction

Data science in Africa is rapidly evolving due to increasing digital connectivity, expanding research initiatives, and a growing demand for data-driven decision-making. According to the 2024 Global System for Mobile Communications Associations (GSMA) report, over 60% (1) of the African population has access to a mobile phone, and internet penetration has reached 27%. The increasing availability of digital systems has created robust infrastructure that enables data to flow across geographic boundaries. Moreover, in the realm of health data, we have witnessed a significant increase in cross-border information flows. The recent COVID-19 pandemic highlighted the importance of cross-border data sharing. In Africa, organizations such as the African Centers for Disease Control and Prevention’s Emergency Operations Center utilize data to monitor public health threats. A recent report showed that the number of health emergencies in Africa surged from 153 outbreaks in 2022–2023 to 242 in 2024, highlighting the need to use data across the 54 member states for pandemic surveillance and response (2). This demonstrates the value of cross-border collaboration in Africa, enabling real-time decision-making to address research and public health priorities by leveraging new skills, such as data science.

In Africa, data science is increasingly transforming health systems by enabling better disease surveillance, personalized care, and policy planning (3). For example, in Nigeria, the *Africa CDC Pathogen Genomics Initiative* has used data science to enhance genomic surveillance for infectious diseases, significantly improving responses to COVID-19 and Lassa fever outbreaks (3–6). In Kenya, the Academic Model Providing Access to Healthcare (AMPATH) uses predictive modeling to optimize HIV care and treatment services, demonstrating how real-time data can inform clinical decision-making and resource allocation (7). At the African Population and Health Research Center, unsupervised machine learning techniques have been used to explore purchasing habits for unhealthy foods in Kenya and to predict cancer survival times (8). South Africa’s National Institute for Communicable Diseases (NICD) also integrates machine learning for tuberculosis tracking and drug resistance modeling (35). Data Science has aided the development of products, including Artificial Intelligence-enabled chatbots, interactive dashboards, healthcare platforms, mobile applications, and web tools (9).

Despite growing interest in data science across Africa, several systemic gaps continue to hinder its effective application, particularly in the health sector. One major challenge is the scarcity of high-quality, interoperable, and real-time data, often due to fragmented data-collection systems and limited investment in data infrastructure (39). The data required for African countries to measure progress toward the SDGs and Agenda 2063 is unprecedented in its scope and granularity (10). For example, 10 African countries, accounting for 19.6% of the continent’s population, have a birth registration system that registers at least 90% of births that occur, while six African countries, accounting for 20.4% of the continent’s population, have less than 30% of births registered (10). There is a need to understand and invest in robust data quality practices to enable better data-driven decision-making and to allow trusted data to drive innovations in health. In parallel, human capacity remains insufficient, with a shortage of trained data scientists, epidemiologists, and data engineers capable of translating complex data into actionable insights (40). A recent World Economic Forum (WEF) report showed that only 1% of the global AI and data science talent pool is based in Africa, highlighting the urgent need for capacity building and training (11). These challenges align with global efforts to adopt FAIR principles (12), which provide a framework for enhancing data utility while respecting sovereignty. However, FAIR adoption in African health research remains uneven due to infrastructure and governance barriers.

This skills gap is exacerbated by limited access to advanced computing infrastructure such as high-performance computing centers, cloud services, and stable internet, especially in rural and underserved regions (4). The GSMA Mobile Economy Report 2023 indicated that while 46% of the African population has access to mobile internet, significant disparities exist between urban and rural areas, affecting the equitable development of data science and AI technologies (13).

Lastly, data governance frameworks and guidelines for adopting new tools such as AI and data science are often weak or inconsistent across countries, leading to uncertainty around data ownership, privacy, and sharing—critical for cross-border collaboration and public trust (6). Despite the milestone in 2023, when 46 African countries adopted the UNESCO Recommendations on Ethics of Artificial Intelligence, several African countries have not fully established or enacted data protection policies, hindering the sharing of data for evidence generation (14).

In light of the identified gaps, we initiated an exploration of the complexities inherent in executing a cross-country data science project in Africa. This initiative is being conducted under the auspices of the Data Science Without Borders project, which is being implemented in Cameroon, Ethiopia, and Senegal. The objectives of the project are threefold: first, to enhance data systems within three selected institutions in the participating countries; second, to assess the human capacity skills required for the effective application of data science and artificial intelligence in generating evidence and addressing national research questions; and third, to implement a data science platform that facilitates evidence generation while maximizing the use of available data sets. This study presents findings from an assessment of the data ecosystem, focusing on the foundational pillars essential to the effective use of data science. Specifically, the evaluation focused on four key areas—data, personnel, infrastructure, and governance—across three project implementation countries.

## Materials and methods

### Ethics statement

The project received ethical approval to conduct the research from Strathmore University (Reference No. *SU-ISERC2367/24*). Furthermore, administrative clearance from each Pathfinder’s administration is required. All methods were performed in accordance with the relevant guidelines and regulations. Written informed consent for participation in the study was obtained voluntarily from the research participants. This was after understanding the study’s purpose. We maintained participants’ confidentiality by keeping identifying information (telephone numbers) under a key and lock, and also by coding using privacy-enhancing methods. The data was analyzed and published in aggregate form to avoid the identification of individual participants. The data is currently stored under key and lock for the 5 years after publication.

### Study areas

Data Science Without Borders (DSWB), inaugurated in February 2024 (15), is a three-year initiative funded by Wellcome (16). It aims to collaboratively design strategies to enhance data systems and the application of data science tools to improve data utilization for evidence generation in Africa. DSWB commenced operations in collaboration with three health institutions, hereinafter referred to as Pathfinders. In Ethiopia, the chosen Pathfinder is the Armauer Hansen Research Institute (AHRI) (17), a medical research institute established in 1970 by the Government of Ethiopia. In Cameroon, the Pathfinder is the Douala General Hospital (DGH) (18), a tertiary public hospital serving approximately 3 million residents and recognized as the country’s largest referral hospital. In Senegal, the Institute for Health Research, Epidemiological Surveillance and Training (IRESSEF) serves as the Pathfinder (19). This non-profit public institution supports public health policies to ensure equitable access to healthcare and significantly contributes to the well-being of populations across Africa. These three purposefully selected health institutions exemplify the primary producers of health data in Africa. This varies from well-established research organizations generating diverse research datasets (AHRI) to hospitals that compile data from electronic hospital records during healthcare delivery (DGH) and public non-profit health institutions engaged in national health initiatives while conducting research aimed at enhancing care and treatment (IRESSEF).

### Study design

We conducted an online cross-sectional quantitative survey between September and October 2024.

### Data collection methods

The semi-structured questionnaire was developed using a structured maturity assessment framework designed to evaluate data capabilities across four key domains: *People and Culture, Data Activities, Business Processes*, and *Technology*. The tool was informed by established data governance and capability maturity models and refined through consultation with co-authors within participating Pathfinder institutions to ensure contextual relevance and clarity. Beyond the maturity domains, there was a deep exploration of the data ecosystem where we explored four main themes: (1) data availability and quality, data collection and storage methods, data analytics approaches, (2) governance, including leadership and policies, (3) data Platform exploring warehousing, security, cloud services, display, and reporting. (4) users—exploring Pathfinder data needs, competence/skills, badging, training, and community of practice of data professionals.

The questionnaire contained 34 structured questions organized across the four domains, each designed to capture different levels of data maturity. Responses followed a five-point ordinal scale (0–4), reflecting progression from limited capability to advanced institutional maturity. Most questions allowed for single or multiple selections depending on the nature of the construct being assessed. Responses were mandatory to ensure complete data collection.

### Sampling procedures and data collection

A purposive sampling approach was employed to identify respondents with specialized expertise in information technology, data science, and data management. These officers could ideally work in their respective roles for at least 2 years, thereby ensuring familiarity with institutional data ecosystems. This sampling strategy ensured that participants possessed the requisite technical and operational understanding of institutional data systems, processes, and governance structures. Data were collected using a semi-structured questionnaire designed in REDCap in English, with a French translation used for data collection. The questionnaire was administered to key informants from the Pathfinder institutions and other purposively selected collaborating organizations across three countries. Each Pathfinder completed the REDCAp questionnaire, as did other purposively selected collaborating institutions in each of the three countries. All data were uploaded to the server at APHRC and are available upon request.

### Data analysis

**Figure 1:**
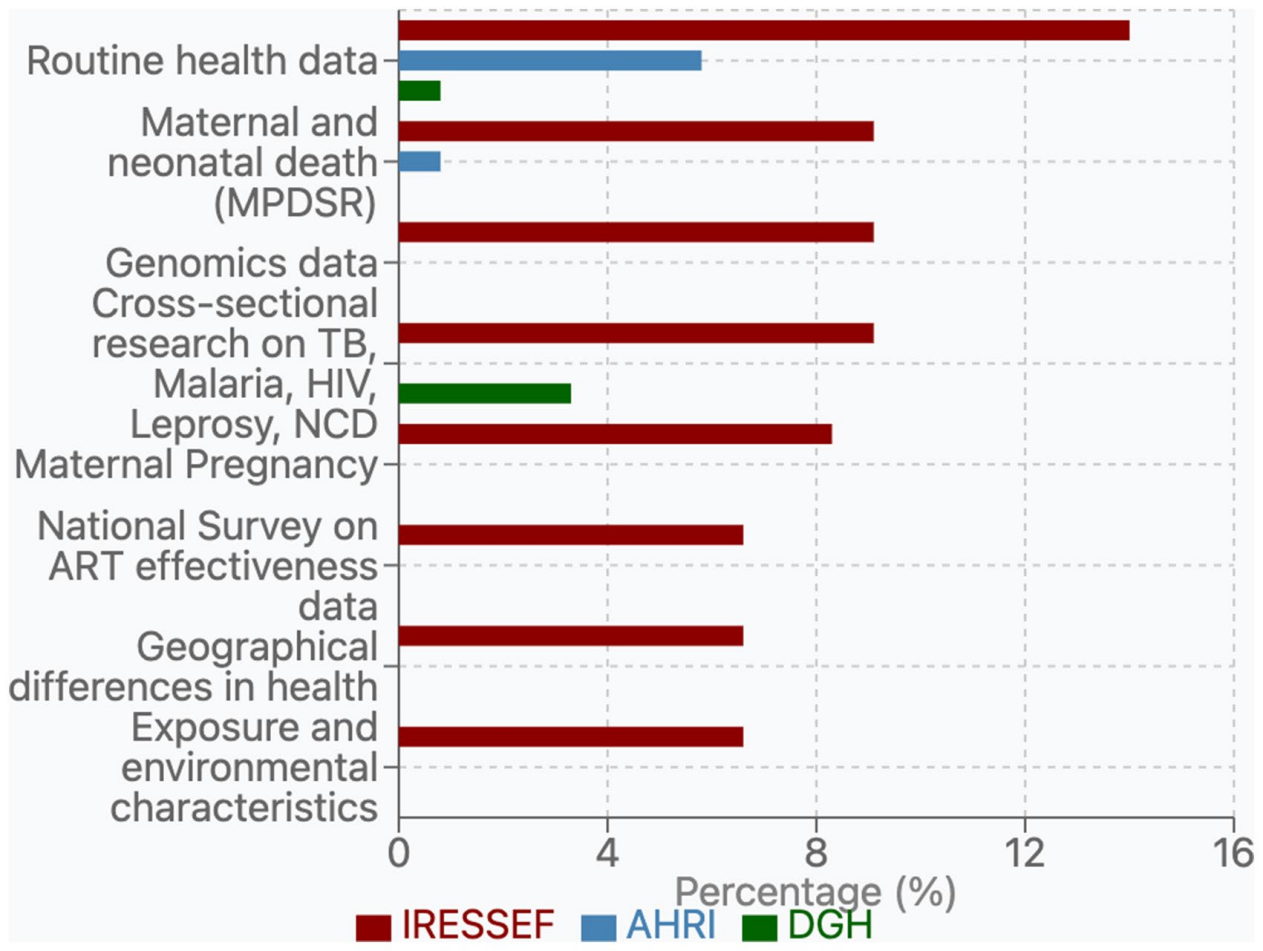
Bar chart displaying percentages of various health data research areas by three organizations: IRESSEF, AHRI, and DGH. IRESSEF shows the highest percentages across most categories.

The data collected through the needs assessment were cleaned, coded, and analyzed using RStudio (version 4.3.1). The responses were reviewed for completeness and consistency before analysis. Variables were categorized according to predefined domains, including data ecosystems, site maturity assessment, training and infrastructure needs. The results were then aggregated by site and summarized into tables and figures to highlight trends and comparative insights across the participating institutions for ease of analysis. Descriptive statistics were used to summarize participating institutions’ demographic and institutional characteristics. Categorical variables were presented as frequencies and percentages, while continuous variables were summarized using means and standard deviations. Cross-tabulations were performed to explore relationships among variables, including institutional characteristics and data governance maturity indicators, current data systems, and institutional needs (Figure 1).

## Results

### Dataset availability across Pathfinder institutions

**Figure 2:**
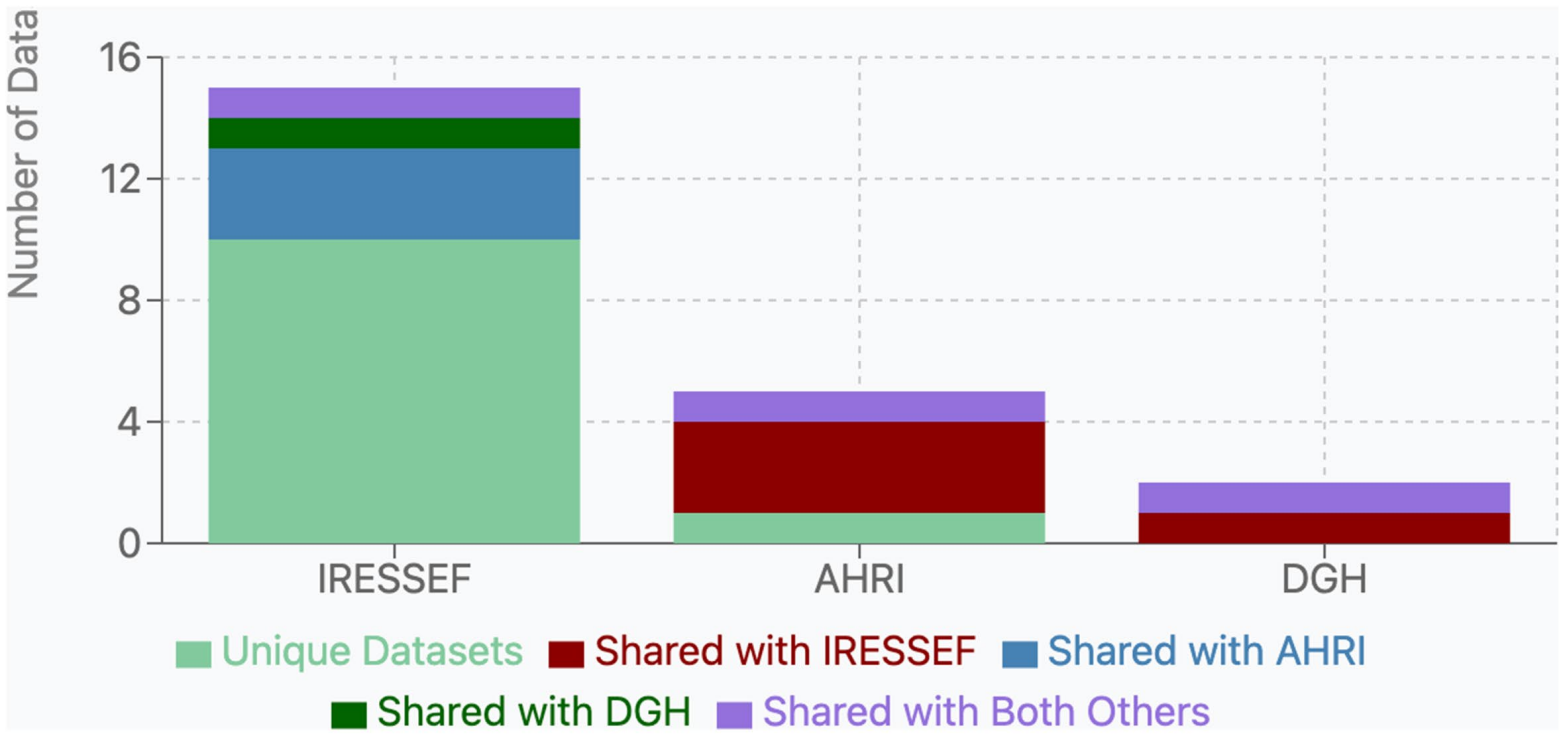
Bar chart showing data sharing among RESSEF, AHRI, and DGH.

**Figure 3:**
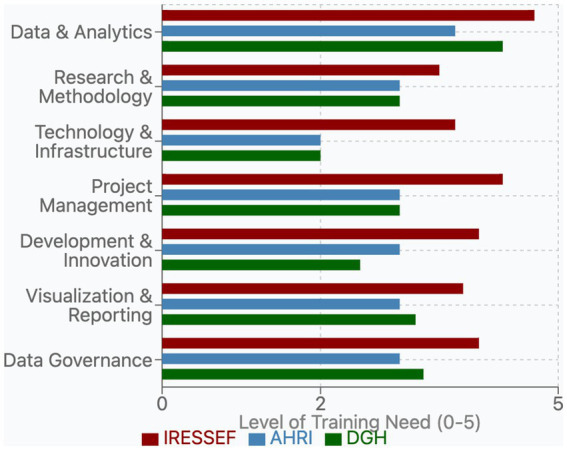
Bar chart showing the level of training need across various categories for three organizations: IRESSEF (red), AHRI (blue), and DGH (green).

Our analysis of public health datasets across the three Pathfinder institutions—IRESSEF, AHRI, and DGH—revealed different patterns of data availability and significant opportunities for collaborative integration (Figure 2 and Table 1). IRESSEF demonstrated the most comprehensive data repository, housing 14 unique dataset types, while AHRI and DGH maintained 5 and 2 dataset types, respectively. Only one dataset category, “Routine health data,” which consists of electronic health service records, was consistently available across all three institutions, highlighting a foundational data resource that could serve as an opportunity for data harmonization, collaboration, and federated analyses. This dataset represented 14.0% of IRESSEF’s data portfolio, 5.8% of AHRI’s, and 0.8% of DGH’s, suggesting varying levels of investment in routine surveillance activities across institutions. The institutions exhibited limited but notable overlap in their datasets. IRESSEF and AHRI shared two dataset categories: “Maternal and neonatal death (MPDSR)” and “Clinical trial sets” data. Similarly, IRESSEF and DGH shared datasets related to “Cross-sectional research on TB, Malaria, HIV, Leprosy, and Non-Communicable Diseases such as Stroke.” No unique dataset categories were shared exclusively between AHRI and DGH, indicating a potential gap in collaborative research between these two institutions. Variability in dataset richness across institutions underscores the need for FAIR-compliant metadata catalogs to enable cross-institutional discovery and reuse (Figure 3).

**Table 1:** FAIR readiness assessment.

FAIR principles	IRESSEF	AHRI	DGH
Findable	Moderate-Data management centralized; multiple digital data sources checked; established focal person and structure for data handling.	Moderate-Centralized data management and designated data focal person; data systems exist but not all sources are integrated.	Low-Moderate-Centralized but smaller data team (3 staff); mixed use of paper/electronic sources, limited standardization.
Accessible	Restricted/Internal Only-Data is stored internally with institutional oversight (IRESSEF server); access managed through principal investigator and legal channels.	Restricted/Internal Only-Access mainly internal within AHRI; data sharing is only through institutional agreements.	Restricted-Data is internal to hospital systems; governed by hospital-level protocols; limited external accessibility.
Interoperable	Partial (CSV/Excel)-They use standard collection tools; some structured formats; limited API integration.	Partial (CSV/Excel)-Common data tools used; structured formats; partial interoperability.	Low (Proprietary or Non-Standardized)-Possible use of mixed tools or local hospital systems; low data exchange capacity.
Reusable	Moderate-Documentation and governance structures exist (through DSWB participation and documentation in eLwazi).	Moderate-Institutional data teams support reuse for internal projects; moderate documentation through DSWB.	Low-Limited metadata or formal documentation; reuse largely dependent on hospital-level permissions and project specific initiatives.

### Institutional dataset specialization

Each institution demonstrated distinct data specializations reflective of their research priorities. IRESSEF maintained a diverse array of unique datasets (10 categories), with particular strengths in Genomics data (9.1%), Maternal Pregnancy (8.3%), and several public health domains, each representing 6.6% of their data portfolio: National Survey on ART effectiveness, Geographical differences in health, and Exposure and environmental characteristics. AHRI exhibited specialized expertise in Surveillance data for malaria, measles, cholera, and meningitis, which constituted 9.9% of their data resources—their most substantial dataset category. AHRI also maintained Basic Research from ongoing projects in health domains, such as leprosy data (0.8%), which was not available at other institutions. DGH, while having fewer unique dataset categories, contributed significantly to cross-sectional research on major infectious diseases, with data on TB, Malaria, HIV, and NCDs representing 3.3% of their data resources.

### Training needs assessment across pathfinder institutions

The survey revealed distinct institutional priorities and significant opportunities for targeted capacity-building for data and other personnel. From a list of over 24 individual training topics reported by the institutions, we categorized them into seven core domains: Data & Analytics, Research & Methodology, Technology & Infrastructure, Project Management, Development & Innovation, Visualization & Reporting, and Data Governance. This framework facilitated a systematic comparison of needs across the three Pathfinders.

### Institutional training profiles

**Figure 4:**
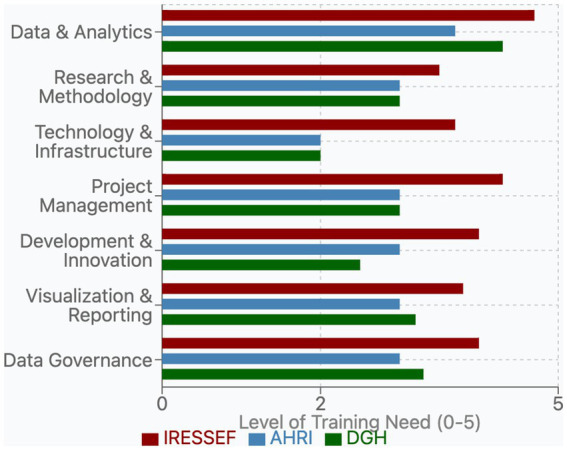
Bar chart showing the level of training need across various skills.

Each institution demonstrated a unique training profile reflective of its current capabilities and strategic objectives. IRESSEF exhibited consistently higher training requirements (average need: 4.1 out of 5) across nearly all domains, with particularly pronounced needs in Data Governance (4.0) and Data & Analytics (4.7). This pattern suggests both ambitious development goals and recognition of critical capacity gaps, especially in data-intensive domains. AHRI displayed moderate, balanced needs (average: 3.0) across most categories, with notable strength in Technology & Infrastructure (2.0) relative to other domains. This profile indicates a more established baseline capacity, with targeted areas for development. DGH presented a variable needs pattern (average: 3.2) characterized by high requirements in specific analytical areas and lower needs in technological domains (Figure 4).

### Priority training needs

Among the listed training gaps, Data Governance emerged as a critical priority domain across Pathfinders, particularly DGH and IRESSEF (Figure 4). Their specific training needs included—Data Protection, Ethical Considerations, and Sharing & Access—reflecting growing recognition of the importance of responsible data stewardship in public health research contexts, which are a bedrock for trustworthy artificial intelligence (7). The assessment revealed substantial variation in institutional capacity within this domain (IRESSEF: 4.0, AHRI: 3.0, DGH: 3.3), highlighting an important area for targeted capacity strengthening. Ethical Considerations, a component of Data Governance, received one of the highest priority ratings from IRESSEF (5.0), underscoring both the importance and perceived capacity gaps in managing ethical dimensions of data utilization within the DSWB consortium and other institutional collaborations. Data Protection similarly showed high priority at IRESSEF (4.0) and DGH (4.0), while receiving moderate attention at AHRI (3.0). Data & Analytics remained a foundational priority area across all Pathfinders (average: 4.7), with need for advanced analysis techniques (such AI and Machine learning) receiving the highest overall training need rating (IRESSEF: 5.0, AHRI: 4.0, DGH: 5.0). This finding aligns with the increasing emphasis on evidence-based decision-making in public health systems globally and reflects the centrality of analytical capabilities at health and research institutions in Africa. Research & Methodology was a critical domain (average: 3.8), with Architecture Integration rated particularly high by IRESSEF (5.0) and DGH (5.0), indicating ongoing efforts to develop integrated research frameworks that will support cross-border evidence generation.

### Infrastructure assessment across the Pathfinder institutions

Our analysis of the existing infrastructure across the three Pathfinder institutions revealed significant disparities in computational resources, connectivity, and service capabilities to support the DSWB project (Table 2). The most pronounced disparity was observed in computing hardware specifications. AHRI reported availability of a server RAM capacity of 512 GB with 64 CPU cores, DGH 32 GB with 2 CPU cores, and IRESSEF 8 GB with 2 CPU cores. These levels of memory capacity could directly impact the institutions’ ability to process large datasets, particularly those required for computationally intensive data science and modeling projects. Storage capacity presented a different pattern, with both AHRI and IRESSEF maintaining equivalent high-capacity storage of 100 TB each, while DGH reported significantly lower storage resources of 1 TB. This pattern suggests that while institutions might invest in data storage infrastructure, they continue to lag in the availability of computational resources to fully leverage these data assets. Similarly, the three Pathfinders demonstrated differences in the availability of services, with only AHRI supporting Windows and Linux environments, while DGH and IRESSEF operate exclusively Windows-based systems. Internet connectivity, a critical factor for collaborative research and data sharing, showed moderate reliability across institutions with periodic outages that are frequent in African settings.

**Table 2:** An assessment of computation infrastructure at the Pathfinder institutions and readiness for data science.

Hardware description	IRESSEF	AHRI	DGH
Availability of servers	Yes	Yes	Yes
Server specs—Memory (RAM)	8GB	512 GB	32GB
Server specs—Processors (CPU)	2 CORES	64 CORES	2 CORES
Server specs—Storage (HD)	100 TB	100 TB	1 TB
Server Operating System (OS)	Windows	Windows, Linux	Windows
Support controlled remote access	Yes	Yes	Yes
Ability of server to support DSWB work	No	Yes	Yes
IT support	Yes	Yes	Yes
Internet Connectivity	Average (Around 5 times black-out a month)	Average (Around 5 times black-out a month)	Good (Less than 4 times a month)
Availability of a dedicated internet services for DSWB work	No	Yes	Yes
Need personal laptops for the data team	Yes	Yes	Yes

## Discussion

Our findings from the baseline needs assessment conducted for collaborating institutions in the Data Science Without Borders Project highlight the state of readiness among health institutions to utilize data science tools for evidence generation. In our study, the three Pathfinder institutions—IRESSEF, AHRI, and DGH—represent diverse sources of health data producers in Africa. Specifically, IRESSEF is a hybrid institution that conducts research while also providing public health care services; AHRI is a pure health research institution; and DGH is a national referral hospital that primarily offers comprehensive health services. Each Pathfinder institution exhibits a different profile when assessed across different dimensions for effective data science implementation. However, our assessment also revealed that data availability alone is an insufficient metric; the quality of the available data—encompassing completeness, accuracy, and consistency—is a critical limiting factor. IRESSEF demonstrates considerable strengths in data availability, particularly in genomics data, and represents institutions where valuable data resources exist but cannot be effectively leveraged due to both infrastructure and skills limitations, as well as uncharacterized data quality. AHRI presents a balanced profile across all dimensions, with moderate data availability, and has particular strengths in surveillance data for infectious diseases. It represents institutions that require further advances and flexibility in implementing analytical solutions. DGH demonstrated limited data availability but has moderately developed infrastructure and targeted training needs. Its data strengths are concentrated in cross-sectional research on infectious diseases. It represents institutions with functional infrastructure but limited data resources and specific skills gaps. Our results contribute to the growing debate on the urgent need for strengthening data systems across Africa (20, 21).

### Opportunities for data integration and sharing

Our assessment identified multiple strategic opportunities for cross-institutional data integration and health information exchange, which are essential for generating continuous, large-scale evidence to enhance public health. For instance, the significant availability of the Routine Health Information System (RHIS) data across all three institutions indicates that this could serve as a foundational element for data harmonization and standardization efforts. Additionally, the availability of unique datasets, such as specific infectious disease surveillance data (e.g., from passive or laboratory-based systems) shared between AHRI and IRESSEF, highlights potential synergies in monitoring infectious diseases. This collaboration may form the basis for coordinated responses to pandemics by sharing these unique datasets. Moreover, IRESSEF and AHRI’s extensive collection of unique datasets, particularly in genomics and environmental health, represents valuable resources that could significantly augment research capabilities across the Pathfinder network, provided that these datasets are standardized and appropriately shared. These findings highlight the current fragmentation of public health data among institutions and underscore the substantial opportunities for strategic integration that will reinforce public health research and data science capabilities. According to Mo Ibrahim, the expected progress and achievement of the Africa Agenda 20,263 will only be realized through unprecedented investments in the scope, granularity, and quality of data, thus emphasizing the need for strong data systems across the continent (10, 22, 23).

### Strategic implications for training for effective data science in African institutions

We further reveal the necessity of building a pool of skilled data staff at the Pathfinders. Our results showed that successful evidence generation and data utilization in African health institutions will depend fundamentally on the availability of skilled data personnel who can address pervasive data quality challenges. Before robust data governance frameworks for ethical sharing can be fully effective, foundational data quality issues must be resolved. These include, but are not limited to, ensuring the completeness, timeliness, accuracy, and consistency of data, as outlined in the WHO Data Quality Assurance framework (24). For instance, in programs requiring concerted patient follow-up like HIV or TB, issues such as lost-to-follow-up, duplicate patient records, and inconsistent lab result reporting can severely compromise the validity of any cross-institutional analysis (25, 26). Therefore, training and capacity building must first equip staff to identify and remediate these issues.

The consistently high ratings for training in analysis techniques (such as machine learning and Artificial Intelligence), architecture integration, data management, and ethical considerations highlighted a clear roadmap for initial training activities that were carried out across the Pathfinder network. The topics covered multiple domains, including data harmonization and standardization, introduction to data science, AI and ethics, and responsible data sharing techniques, with a renewed emphasis on practical data quality assessment and improvement methods. These trainings paved the way for the creation of an integrated approach to health data utilization across the three institutions, balancing analytical capabilities with a foundational commitment to data quality, appropriate governance and health information exchange frameworks (21).

Our findings further underscore that capacity-building efforts must address both skills development and infrastructure enhancement concurrently. Training in advanced analytical techniques or data governance frameworks may yield limited practical impact without corresponding improvements in computational resources and service capabilities. While different institutions have varying available datasets and moderate training needs, the substantial hardware limitations may significantly constrain their ability to implement AI and machine learning analytical approaches for evidence generation (27–29).

Platforms such as the INSPIRE Network (30), the Africa Population Cohort Consortium (31), the DSI Africa collaborations and key national centers such as the Centre for Epidemiological Modeling and Analysis (CEMA) at the University of Nairobi (32), facilitate access to data sets at the national and multi-country levels. Investment in such platforms is essential for advancing research efforts across the continent to promote synchronized investment in data systems (12, 33, 34). Additionally, ensuring data in Africa is findable, accessible, interoperable, and reusable (FAIR) will promote the culture of data sharing and collaborative data projects (27). Although this baseline needs assessment did not explore in depth the platforms hosted by each Pathfinder organization, future papers will cover this important aspect. This will help to understand existing research Information Technology maturity models, which count on investments in research platforms for clinical trials, survey research, observational research and translational research.

The urgent need to strengthen data governance systems in Africa is another important pillar. This study found few institutions with supportive policies regulating data access, utilization, and protection (22). This needs to be strengthened in many African countries. There is a need to institute data-sharing agreements, policies, and laws that ensure equitable and ethical access, and utilization of the datasets (37). Although there is wide adoption of ethical requirements for obtaining approvals for studies in the medical, natural, and biological sciences domains, this only addresses the ethical aspects. The idea of data access policies remains gray, leaving gaps for continued siloed working and hiding of data (37). Such a system would enable universities and research agencies to use the available datasets and then develop products like Patient Chatbots, and predictive analytical models that policymakers could then use for decision-making (29). The average evidence has demonstrated that having data alone without policies, agreements, and governance/structures on how to access it is close to useless. Why? This is because a lot of datasets could be generated (even with their weaknesses of incompleteness and inaccuracies), but if not accessed by those who can derive value from them, then they are useless (36). The challenges of data sharing are exacerbated by varying advancements in cross-border data sharing and the development of Data Protection and Privacy Regulation (38).

Advancements to new forms for collaborating while respecting boundaries of data collection, will be critical for collaboration across the continent. Without robust data governance frameworks in Africa, investments in data systems, analytical capabilities, or infrastructure may yield limited benefits or introduce new risks. The forms of data governance must be matched with robust infrastructure, as it remains a rate-limiting factor for effective advancement in data science and AI deployment.

Our study has identified several next steps that guided the project implementation. The team has adopted a phased implementation approach, recognizing that addressing data, capacity, governance, and infrastructure gaps cannot be done simultaneously. The following phases were agreed upon and included: Phase 1, establishing data governance frameworks and addressing critical infrastructure gaps; Phase 2, implementing core training programs in high-priority domains; Phase 3, developing advanced analytical capabilities and cross-institutional integration; and Phase 4, deploying federated platforms for pooled analysis across the consortium. This will allow staged customization of project implementation objectives, dependent on the maturity of the data ecosystems.

### The foundations for transformative collaboration

Our assessment highlights the challenges and opportunities in health data science across Africa, noting disparities in infrastructure, training, and data resources. Instead of viewing these differences as obstacles, they should be seen as a basis for collaborative partnerships that leverage each institution’s strengths. Institutions can contribute unique components to a collective data ecosystem, creating a model for pan-African collaboration that values diversity and enhances overall capacity beyond what any single institution could achieve alone.

### Moving beyond traditional capacity building

The findings challenge conventional capacity building methods that focus on standardized training or infrastructure development. These methods overlook the complex interdependencies between data resources, governance, human capabilities, and technical infrastructure. Effective capacity building must be contextual and integrated, addressing all dimensions while leveraging existing institutional strengths. Our results suggest that data science initiatives in Africa should adopt a “network of excellence” model, distributing capabilities across institutions and ensuring seamless integration and AI deployment.

### Data governance as the critical foundation

The key finding from our assessment is that data governance is crucial for effective collaboration. The need for training in data sharing and governance highlights a critical gap that must be addressed. Without strong frameworks for data ownership, sharing, security, and ethical use, advancements may yield limited benefits or introduce new risks. This aligns with broader concerns in Africa about data sovereignty and the protection of sensitive health information. By prioritizing data governance, Pathfinder institutions can develop models that facilitate productive data sharing while maintaining control over their resources, offering templates for other African institutions involved in data science.

### A federated approach to African data science and transfer learning

The resource disparities identified in our assessment, especially regarding computational infrastructure, indicate that a federated approach to data science is ideal for African contexts. Instead of requiring each institution to develop independent capabilities—both financially and technically challenging—a federated model distributes functions across a network to ensure equitable benefits for all partners. This model could expand to various African institutions, establishing nodes as centers of excellence in areas like data curation, computational analysis, governance, or training. It also aligns with global best practices in health data science, emphasizing the importance of analyzing data in diverse contexts while respecting governance boundaries.

### Building sustainable human capacity

Our assessment of training needs highlights that sustainable data science initiatives require not only technical skills but also leadership, collaboration, and ethical considerations. This suggests that training programs and curricula across Africa must integrate technical expertise with contextual knowledge and ethical reasoning. By adopting these comprehensive approaches, African institutions can create educational models tailored to the continent’s unique needs.

### Study limitations

While our comprehensive assessment provided valuable insights into data availability and governance, training needs, and infrastructure capabilities across the three Pathfinder institutions, several limitations should be acknowledged when interpreting these findings and applying the recommendations. Our assessment, while multidimensional, was limited in scope to three specific institutions (IRESSEF, AHRI, and DGH) and may not capture the full range of variation present across African research institutions. The selected Pathfinder institutions, while representing different regions and contexts, cannot fully represent the diversity of research environments across the continent. Furthermore, the depth of assessment within each dimension was constrained by practical considerations of time and resources, potentially omitting nuanced aspects of institutional capabilities. The evaluation relied on self-reported data, which could have been influenced by subjective perceptions and varying levels of self-awareness regarding institutional capabilities. In addition, the perspectives shared by institutional leadership and technical staff may have underrepresented those of end-users of data systems, researchers, and external collaborators who were not interviewed. Lastly, the assessment represents a snapshot at a particular point in time at project inception within rapidly evolving institutions. The dynamic nature of technological infrastructure, staffing, data literacy, and resources means that some findings may have become outdated relatively quickly. To address these limitations, the DSWB project will conduct a repeat survey toward project closure to assess changes in capabilities and the decommissioning institutional development trajectories and the impact of the project interventions. Where possible, we will include broader institutional sampling to expand the assessment to a wider range of African institutions, thereby capturing greater contextual diversity. Lastly, we plan to incorporate perspectives from a range of stakeholders, including community representatives, policymakers, and international partners. Despite these limitations, our multidimensional assessment provides a valuable foundation for understanding the current landscape of data science capabilities across the Pathfinder institutions and developing contextually appropriate strategies for enhancement. The limitations identified should be viewed not as undermining the validity of the findings but as defining important boundaries for their interpretation and application while pointing toward opportunities for further research and refinement.

## Conclusion

The insights from our comprehensive assessment of the Pathfinder institutions illuminate a path toward more effective, equitable, and sustainable data science collaboration across Africa. By embracing institutional diversity, prioritizing data governance, adopting federated approaches, developing integrated human capacity, and implementing phased development, similar initiatives can navigate the complex landscape of African data science more effectively. A FAIR-guided federated model can balance data sovereignty with collaborative analysis, offering a blueprint for equitable data science in Africa. These findings suggest that the future of health data science in Africa lies not in isolated centers of excellence but in collaborative networks that distribute capabilities across institutions, ensuring all partners contribute to and benefit from the collective resources. The Pathfinder institutions—with their diverse profiles and complementary strengths—provide a microcosm of the broader African context and a model for how distributed excellence can yield collective impact. As data science becomes increasingly central to addressing the continent’s public health challenges, the lessons from this assessment offer valuable guidance for building collaborative ecosystems that are technically robust, ethically sound, and uniquely adapted to African contexts and priorities. By building on these insights, African institutions can develop approaches to data science that not only address local needs but also contribute varying perspectives and methodologies to the global data science community.

## Data Availability

The datasets presented in this study can be found in online repositories. The names of the repository/repositories and accession number(s) can be found in the article/supplementary material.
